# A Matrix Prediction Model for the 6-Month Mortality Risk in Patients With Anti-Melanoma Differentiation-Associated Protein-5-Positive Dermatomyositis

**DOI:** 10.3389/fmed.2022.860798

**Published:** 2022-04-01

**Authors:** Zhi-Ming Ouyang, Jian-Zi Lin, Ao-Juan Tang, Ze-Hong Yang, Li-Juan Yang, Xiu-Ning Wei, Qian-Hua Li, Jin-Jian Liang, Dong-Hui Zheng, Bing-Peng Guo, Gui Zhao, Qian Han, Lie Dai, Ying-Qian Mo

**Affiliations:** ^1^Department of Rheumatology, Sun Yat-sen Memorial Hospital, Sun Yat-sen University, Guangzhou, China; ^2^Department of Rheumatology, Shenshan Medical Center, Memorial Hospital of Sun Yat-sen University, Shanwei, China; ^3^Departments of Radiology, Sun Yat-sen Memorial Hospital, Sun Yat-sen University, Guangzhou, China; ^4^State Key Laboratory of Respiratory Disease, National Clinical Center for Respiratory Disease, Guangzhou Institute of Respiratory Health, The First Affiliated Hospital of Guangzhou Medical University, Guangzhou, China

**Keywords:** anti-melanoma differentiation-associated protein-5, dermatomyositis, mortality, fever, carcinoma embryonic antigen, serum ferritin, matrix prediction model

## Abstract

**Objectives:**

The purpose of this study was to investigate the baseline independent risk factors for predicting 6-month mortality of patients with anti-melanoma differentiation-associated gene 5 (anti-MDA5)-positive dermatomyositis (DM) and develop a matrix prediction model formed by these risk factors.

**Methods:**

The hospitalized patients with DM who completed at least 6-month follow-up were recruited as a derivation cohort. The primary exposure was defined as positive anti-MDA5 at the baseline. The primary outcome was all-cause 6-month mortality after enrollment. A matrix prediction model was developed in the derivation cohort, and another published cohort was used for external validation.

**Results:**

In derivation cohort, 82 patients with DM were enrolled (mean age of onset 50 ± 11 years and 63% women), with 40 (49%) showing positive anti-MDA5. Gottron sign/papules (OR: 5.135, 95%CI: 1.489–17.708), arthritis (OR: 5.184, 95%CI: 1.455–18.467), interstitial lung disease (OR: 7.034, 95%CI: 1.157–42.785), and higher level of C4 (OR: 1.010, 95%CI: 1.002–1.017) were the independent associators with positive anti-MDA5 in patients with DM. Patients with anti-MDA5-positive DM had significant higher 6-month all-cause mortality than those with anti-MDA5-negative (30 vs. 0%). Among the patients with anti-MDA5-positive DM, compared to the survivors, non-survivors had significantly advanced age of onset (59 ± 6 years vs. 46 ± 9 years), higher rates of fever (75 vs. 18%), positive carcinoma embryonic antigen (CEA, 75 vs. 14%), higher level of ferritin (median 2,858 ug/L *vs*. 619 ug/L, all *p* < 0.05). A stepwise multivariate Cox regression showed that ferritin ≥1,250 μg/L (HR: 10.4, 95%CI: 1.8–59.9), fever (HR: 11.2, 95%CI: 2.5–49.9), and positive CEA (HR: 5.2, 95%CI: 1.0–25.7) were the independent risk factors of 6-month mortality. A matrix prediction model was built to stratify patients with anti-MDA5-positive DM into different subgroups with various probabilities of 6-month mortality risk. In an external validation cohort, the observed 6-month all-cause mortality was 78% in high-risk group, 43% in moderate-risk group, and 25% in low-risk group, which shows good accuracy of the model.

**Conclusion:**

Baseline characteristics such as fever, ferritin ≥1,250 μg/L, and positive CEA are the independent risk factors for 6-month all-cause mortality in patients with anti-MDA5-positive DM. A novel matrix prediction model composed of these three clinical indicators is first proposed to provide a chance for the exploration of individual treatment strategies in anti-MDA5-positive DM subgroups with various probabilities of mortality risk.

## Highlights

- The risk factors for 6-month all-cause mortality in patients with anti-MDA5-positive dermatomyositis (DM) vary among different studies and have not been determined. This study first described positive carcinoma embryonic antigen (CEA) at the baseline as an independent risk factor for death within 6 months in patients with anti-MDA5-positive DM.
- Other than the radiomic models which need the intricate algorithm, we proposed a novel matrix prediction model built based on three clinical characteristics (fever, serum ferritin, and CEA) which could be obtained more conveniently in clinical practice. The application of baseline characteristics as mortality predictors can forecast the 6-month mortality once the diagnosis of anti-MDA5-positive DM before the therapeutic options made in clinical practice.
- Different from previously published models, the matrix model can provide the mortality risk probability in any combination of three features, which makes the prediction more accessible to clinicians and simpler to spread in clinical practice. This model is helpful for the risk stratification of patients with anti-MDA5-positive DM and provides a chance for the exploration of individual treatment strategies in subgroups with various degrees of mortality risk.

## Introduction

Idiopathic inflammatory myopathy (IIM) is a heterogeneous family of autoimmune disorders usually characterized by varying degrees of chronic muscle inflammation with shared clinical manifestations but different treatment responses and prognoses (1). Besides the muscle symptoms, extra-muscular manifestations, such as skin rash, arthritis, and interstitial lung disease (ILD), are common, which emphasizes the systemic inflammatory nature of these disorders (1). Along with the discovery of myositis-specific antibodies, IIM is categorized into not only two classical subgroups such as dermatomyositis (DM) and polymyositis (PM), but also two new subgroups with distinct clinical manifestations and muscle histopathological features, which are anti-synthetase syndrome and immune-mediated necrotizing myopathy (1–3). According to the 239th European NeuroMuscular Center classification criteria for DM, patients with anti-synthetase autoantibodies will be classified as having the anti-synthetase syndrome but not DM (4).

Besides anti-synthetase autoantibodies, the autoantibody recognizing melanoma differentiation-associated gene 5 (anti-MDA5) is of the greatest concern as a DM-specific autoantibody, since it delineates a unique clinical phenotype of DM with a high risk of life-threatening lung complications but the signs of myositis are mild or absent (5). Of note, in terms of impressively high mortality, compared to all other forms of myositis, anti-MDA5-positive DM remains the most intractable and challenging. Unfortunately, most fatalities occur within the first half-year of the disease despite intensive immunosuppressive treatment, which leads to high 6-month mortality reports varying from 33 to 66% according to the different cohort studies worldwide (6–11).

Emerging cohort studies tried to seek clinical features to predict the 6-month mortality risk of patients with anti-MDA5-positive DM (9–15). The poor survival rate of anti-MDA5-positive DM could be explained by the rapidly progressive ILD (RP-ILD), which is definitely a risk factor for death in patients with anti-MDA5-positive DM. The most striking results were from a recent study that quantitatively assessed pulmonary high-resolution computed tomography (HRCT) images by radiomic approach in deep learning algorithm (10). The applied radiomic score, forced vital capacity percentage of predicted (FVC%), and age were combined to establish a multidimensional risk prediction model for the 6-month mortality in anti-MDA5-positive with ILD (10). However, the intricate algorithm of radiomic score hinders its application in most medical facilities. An HRCT imaging score (HRCT score) which belonged to a composite risk score can predict the mortality in patients with amyopathic DM with ILD (15), but it was also too complex to apply in routine clinical practice. Referring to the respiratory physiological parameters, the ventilation dysfunction (FVC% <50%) independently predicted the 6-month all-cause mortality in patients with anti-MDA5-positive DM, but the air exchange dysfunction (e.g., carbon monoxide diffusion) as an early feature of ILD was not reported as a risk factor of mortality (9). Pulmonary function tests are still far away from standardization because of inter-equipment bias, inter-technician bias, and the tolerance of patients.

Considering the difficulty in popularizing the above HRCT-based or FVC%-based predictors, routine clinical characteristics at baseline are still desired candidates for screening potential mortality predictors, to as early as possible stratify the mortality risk in patients with anti-MDA5-positive DM before making therapeutic strategies. Recently, the Multicenter Retrospective Cohort of Japanese Patients with Myositis-Associated ILD (JAMI) Investigators reported a matrix model for risk prediction using CRP and KL-6 combined with anti-MDA5, which might be useful for predicting prognosis in patients with PM/DM-ILD (16). But this model excluded some biomarkers such as serum ferritin since it was significant for only patients with anti-MDA5-positive rather than all patients with PM or DM. To exclusively build a matrix prediction model formed by baseline characteristics for 6-month mortality risk of patients with anti-MDA5-positive DM, we prospectively enrolled a real-world observational cohort of patients with DM for derivation. First, patients with anti-MDA5-positive DM were divided into non-survivors and survivors based on the outcome within 6-month follow-up. We delineated significantly differential characteristics between non-survivors and survivors, among which the independent risk factors which can predict all-cause death within six months were identified by multivariate Cox regression analyses. Next, we used these risk factors to build an applicable matrix prediction model to stratify patients with anti-MDA5-positive DM into high risk, moderate risk, or low risk for death within 6 months. Finally, we validated this model in another independent cohort.

## Materials and Methods

### Study Design and Patient Recruitment

This was a real-world prospective observational study. The hospitalized patients with DM at the Department of Rheumatology, Sun Yat-sen Memorial Hospital, Sun Yat-Sen University, Guangzhou, P.R China were recruited from January 2018 to February 2021 as a derivation cohort. Consecutive patients with DM were included if they fulfilled two criteria including (i) aged above 18 years; (ii) diagnosed as having DM according to the criteria proposed by Bohan and Peter (17) or the modified Sontheimer definitions (18). The exclusion criteria included (i) fulfilling the classified criteria of anti-synthetase syndrome, PM, immune-mediated necrotizing myopathy, or other connective tissue diseases; (ii) complicated with lethal carcinoma; (iii) hospitalizing due to other disease conditions; (iv) incomplete clinical or laboratory data; (v) not completed at least 6-month follow-up. This study was conducted in compliance with the Declaration of Helsinki and the protocol was approved by the Medical Ethics Committee of Sun Yat-sen Memorial Hospital (SYSEC-KY-KS-2021-248). All participants gave their written informed consent before data collection. A published cohort of patients with anti-MDA5-positive DM from another institution (the first affiliated hospital of Guangzhou Medical University, Guangzhou, P.R China) which had the similar inclusive criteria and exclusive criteria was used for the external validation (9, 10).

### Data Collection at Enrollment

Demographic and clinical data were collected at enrollment. Clinical data included (i) general assessments: fever (axillary temperature ≥37°C), arthritis, and so on; (ii) skin signs: mechanic's hands, Gottron sign/papules, heliotrope rash, V-like sign, and shawl sign; (iii) muscle assessments: muscle weakness and pain, difficulty swallowing, electromyography, and muscle biopsy. The diagnosis of clinically amyopathic dermatomyositis (CADM) referred to Sontheimer's provisional CADM criteria (18); (iv) pulmonary assessments: cough, dyspnea, and pulmonary HRCT; the classification of ILD (19) and HRCT score (15) was assessed by the experienced radiologists who were blinded to clinical data; (v) laboratory examinations: serum albumin (ALB), muscle enzymes [such as creatine kinase (CK), lactate dehydrogenase (LDH), alanine transaminase (ALT), and aspartate transaminase (AST)], serum ferritin (chemiluminescence method, normal range 28–365 ng/ml), and so on, and (vi) cancer screening: systemic symptoms, physical examinations, serum cancer biomarkers such as carcinoma embryonic antigen (CEA, electrochemiluminescence method, normal range ≤ 5 ng/ml), the corresponding ultrasonography or radiographic computed tomography (CT)–magnetic resonance imaging (MRI) or even positron emission tomography CT (PET-CT) examinations, various endoscopic examinations if necessary.

### Exposure

The primary exposure was defined as patients with DM exposed to anti-MDA5 (patients with anti-MDA5-positive DM) or not (patients with anti-MDA5-negative DM). The anti-MDA5 was qualitatively determined by line immunoassay testing (D-tek s.a., Rue René Descartes, Belgium) and was contemporarily quantitatively determined by the enzyme-linked immunosorbent testing (Euroimmun AG. Lubeck, Germany).

### Follow-Up

All patients were treated according to the guidelines of recommendations for patients with DM (6, 20). The clinical evaluation and therapeutic regimens were recorded during the 6-month follow-up period without interference with physicians' therapeutic strategies. Therapeutic regimens included the usage and dosage of glucocorticoids, cyclophosphamide, mycophenolate mofetil, Janus kinase (JAK) inhibitors including baricitinib and tofacitinib, calcineurin inhibitors, sulfamethoxazole (SMZ)/trimethoprim (TMP), therapeutic or preventive antibiotic, antiviral, intravenous immunoglobulin (IVIG), and anti-fibrosis therapy at each visit. Physical examinations and laboratory examinations were conducted at each visit. Pulmonary assessments and monitoring of cancer or infection were performed during the follow-up. RP-ILD was defined as either worsening of dyspnea and CT progression within 1 month, or deterioration to respiratory failure within 3 months since the onset of respiratory symptoms (21, 22).

### Outcome

The primary outcome was 6-month all-cause mortality of patients with DM since enrollment.

### Statistical Analysis

IBM SPSS Statistics software for Windows version 25.0 (IBM, Armonk, NY, USA) was used for statistical analyses. Values of continuous variables were presented as mean and standard deviation (SD) or median with inter-quartile range (IQR) according to the distributions. Totally, two independent samples *t*-test or the Mann–Whitney *U* test were used for comparison between two independent groups, and one-way analysis of variance (ANOVA) or Kruskal–Wallis analysis of variance on ranks were used among three or more groups according to the distributions. Categorical variables were presented as numbers and percentages. Chi-square test or Fisher's exact test was used to compare categorical variables. Bonferroni correction was used for multiple comparisons in three or more groups.

In derivation cohort, logistic regression analyses by calculating odds ratio (OR) and 95% confidence interval (CI) were used to identify potential associated factors with positive MDA5 in patients with DM. The predictive accuracy of baseline characteristics for distinguishing two subgroups was assessed by receiver operating characteristic (ROC) curve analysis with the area under the curve (AUC). Multivariate Cox regression analyses by calculating hazard ratio (HR) and 95% CI were used to identify potential associated risk factors for all-cause death within 6 months in patients with anti-MDA5-positive DM and to adjust the potential confounders. Furtherly, a matrix prediction model was built and R software was used to calculate the mortality risk probability and 95% CI in each matrix. The patients with anti-MDA5-positive DM were stratified into three subgroups: high risk (the estimated mortality >50%), moderate risk (the estimated mortality between 49 and 10%), and low risk (the estimated mortality <10%). Then, this matrix prediction model was validated in an external cohort. All significance tests were two-tailed and were conducted at the 5% significance level.

## Results

### Clinical Features of Patients With DM in Derivation Cohort

**Figure 1:**
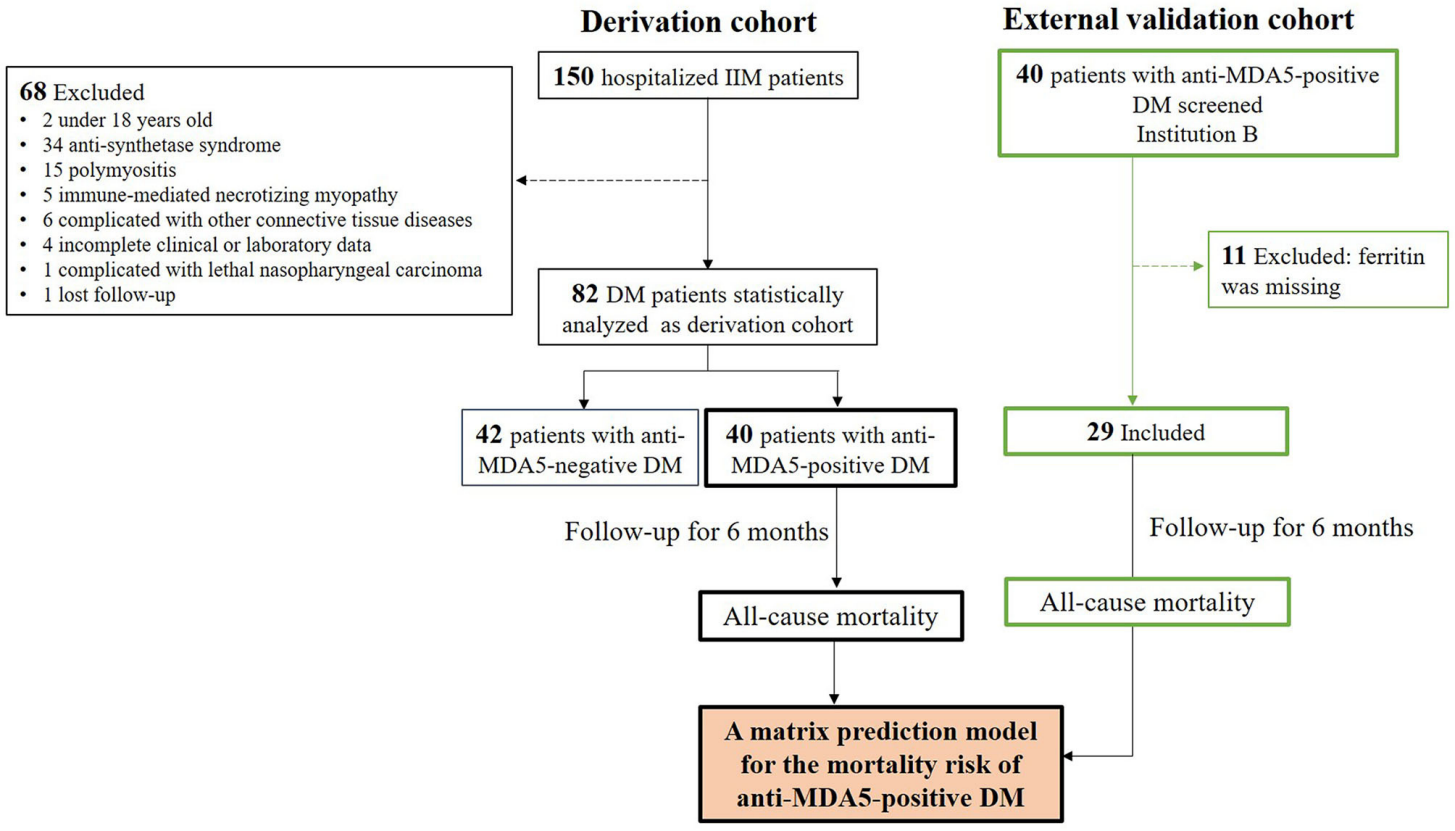
Flow diagram of patients with DM during 6-month follow-up. The derivation cohort was used to develop a matrix prediction model, and external validation cohort from another institution was used to validate the model. IIM, Idiopathic inflammatory myopathy; DM, dermatomyositis.

Among 150 hospitalized patients with IIM during the enrollment period, 82 patients with DM who fulfilled the inclusion criteria and did not fulfill the exclusive criteria were included for statistical analysis (Figure 1). As shown in Table 1, there were 52 (63%) female patients with DM, with age 50 ± 11 years of onset, 7 ± 11-month disease course, and 13 (16%) with smoking. All patients showed DM-specific skin signs, and the most frequent signs were Gottron sign/papules (57%, 47/82) and mechanic's hands (45%, 37/82). The most common muscle symptom was muscle weakness (54%, 44/82), and 33 (40%) patients with DM were considered as CADM. There were 79% of patients complicated with ILD, while the frequent pattern was nonspecific interstitial pneumonia (NSIP) including pure NSIP (49%, 40/82) and combined with organizing pneumonia (OP, 16%, 13/82). A total of 18 (22%) patients with DM presented with RP-ILD during the follow-up. Additionally, 16 (20%) patients with DM had positive CEA. The median value of positive CEA was 11.6 ng/ml, with a maximum of 22.0 ng/ml. No definite neoplastic lesion was found during the follow-up.

**Table 1:** Clinical characteristics of 82 patients with DM and the subgroups based on the anti-MDA5.

**Characteristics**		**Patients with DM**		
	**Patients with DM** **(***n*** = 82)**	**Anti-MDA5-Positive** **(***n*** = 40)**	**Anti-MDA5-Negative** **(***n*** = 42)**	***p***
**Demographic**				
Female, *n* (%)	52 (63%)	25 (63%)	27 (64%)	0.867
Age of onset (years), $\bar{x}$ ± s	50 ± 11	50 ± 10	50 ± 12	0.783
Disease course (months), $\bar{x}$ ± s	7 ± 11	4 ± 7	11 ± 12	0.001
Smoking, *n* (%)	13 (16%)	7 (18%)	6 (14%)	0.690
**General assessments**				
Fever, *n* (%)	26 (32%)	14 (35%)	12 (29%)	0.532
Arthritis, *n* (%)	44 (54%)	31 (78%)	13 (31%)	<0.001
**Skin signs**				
Mechanic's hands, *n* (%)	37 (45%)	21 (53%)	16 (38%)	0.190
Gottron sign/papules, *n* (%)	47 (57%)	31 (78%)	16 (38%)	<0.001
Heliotrope rash, *n* (%)	18 (22%)	6 (15%)	12 (29%)	0.138
V-like sign, *n* (%)	29 (35%)	16 (40%)	13 (31%)	0.392
Shawl sign, *n* (%)	19 (23%)	12 (30%)	7 (17%)	0.153
**Muscle assessments**				
Muscle weakness, *n* (%)	44 (54%)	19 (48%)	25 (60%)	0.275
Muscle pain, *n* (%)	29 (35%)	14 (35%)	15 (36%)	0.946
Difficulty swallowing, *n* (%)	16 (20%)	6 (15%)	10 (24%)	0.314
CADM, *n* (%)	33 (40%)	18 (45%)	15 (36%)	0.391
**Pulmonary assessments**				
Cough, *n* (%)	32 (39%)	15 (38%)	17 (40%)	0.782
Dyspnea, *n* (%)	30 (37%)	15 (38%)	15 (36%)	0.867
ILD, *n* (%)	65 (79%)	38 (95%)	27 (64%)	0.001
NSIP, *n* (%)	40 (49%)	18 (45%)	22 (52%)	0.504
OP, *n* (%)	7 (9%)	6 (15%)	1 (2%)	0.099
NSIP+OP, *n* (%)	13 (16%)	10 (25%)	3 (7%)	0.027
UIP, *n* (%)	2 (2%)	1 (3%)	1 (2%)	1.000
AIP, *n* (%)	3 (4%)	3 (8%)	0	0.223
RP-ILD, *n* (%)	18 (22%)	14 (35%)	4 (10%)	0.005
**Laboratory examinations**				
ALB (g/L), median (IQR)	32 [26–35]	31 [26–33]	33 [28–37]	0.051
CK (U/L), median (IQR)	94 [41–315]	67 [36–135]	136 [72–1,077]	0.001
LDH (U/L), median (IQR)	320 [264–481]	314 [269–455]	324 [254–528]	0.587
ALT (U/L), median (IQR)	37 [20–59]	36 [19–64]	38 [20–59]	0.838
AST (U/L), median (IQR)	39 [23–96]	42 [23–93]	37 [22–98]	0.845
CRP (mg/dL), median (IQR)	0.5 [0.3–1.7]	0.5 [0.3–1.8]	0.4 [0.3–1.6]	0.592
Ferritin (ug/L), median (IQR)	603 [251–1,251]	719 [379–2,305]	474 [222–954]	0.031
KL-6^▴^ (U/ml), median (IQR)	1057 [647–1,790]	1315 [889–1,754]	1318 [400–2,305]	0.693
Positive CEA, *n* (%)	16 (20%)	13 (33%)	3 (7%)	0.004

▴ *The Krebs von den Lungen-6 (KL-6) was tested in 16 patients with anti-MDA5-positive DM and 7 patients with anti-MDA5-negative DM, a total of 23 patients with DM*.

*MDA5, melanoma differentiation-associated protein-5; DM, dermatomyositis; CADM, clinically amyopathic dermatomyositis; ILD, interstitial lung disease; NSIP, nonspecific interstitial pneumonia; OP, organizing pneumonia; UIP, usual interstitial pneumonia; AIP, acute interstitial pneumonia; RP-ILD, rapidly progressive ILD; ALB, albumin; CK, creatine kinase; LDH, lactate dehydrogenase; ALT, alanine aminotransferase; AST, aspartate aminotransferase; CRP, C-reactive protein; CEA, carcinoembryonic antigen; IQR, interquartile range*.

### Clinical Features of Patients With Anti-MDA5-Positive DM

According to the primary exposure, patients with DM were divided into anti-MDA5-positive group (*n* = 40, 49%) and anti-MDA5-negative group (*n* = 42). There were 18 (45%) patients with CADM in patients with anti-MDA5-positive DM, and 15 (36%) patients with CADM in patients with anti-MDA5-negative DM (*p* > 0.05).

Compared with the anti-MDA5-negative group, patients with anti-MDA5-positive DM had shorter disease courses from the disease onset to enrollment (4 ± 7 vs. 11 ± 12 months), higher rates of arthritis (78 vs. 31%) and Gottron sign/papules (78 vs. 38%), lower level of CK (median 67U/L vs. 136U/L), higher level of serum ferritin (median 719 vs. 474 ug/L), and C4 (median 289 *vs*. 232 mg/L), higher rate of positive CEA (33 vs. 7%, all *p* < 0.05, Table 1 and Supplementary Table 1). The rate of ILD in patients with anti-MDA5-positive DM was significantly higher than the anti-MDA5-negative group (95 vs. 64%, *p* = 0.001). Although there was no difference in the constituent ratio of pure NSIP between two groups, the constituent ratio of NSIP combined with OP (NSIP+OP) in the patients with anti-MDA5-positive DM was significantly higher than that in the negative group (25 vs. 7%, *p* = 0.027). Also, the rate of RP-ILD was significantly higher in patients with anti-MDA5-positive DM (35 vs. 10%, *p* = 0.005). Univariate and multivariate logistic regression analyses showed Gottron sign/papules (OR: 5.135, 95%CI: 1.489–17.708), arthritis (OR: 5.184, 95%CI: 1.455–18.467), ILD (OR: 7.034, 95%CI: 1.157–42.785), and higher level of C4 (OR: 1.010, 95%CI: 1.002–1.017) were the independent associators with positive anti-MDA5 in patients with DM (Supplementary Table 2).

### The Medications and Outcome of Patients With Anti-MDA5-Positive DM

The medications of patients with anti-MDA5-positive DM during 6-month follow-up are shown in Table 2. Up to 90% (*n* = 36) of patients with anti-MDA5-positive DM received prednisone equivalent 1–2 mg/kg treatment. The most commonly used immunosuppressant was cyclophosphamide (73%, 29/40), next was calcineurin inhibitors (43%, 17/40), while only 15% of patients received JAK inhibitors. According to the primary outcome, the 6-month all-cause mortality was 30% in 40 patients with anti-MDA5-positive DM, which was significantly higher than 0% in the anti-MDA5-negative group (*p* < 0.001). A total of eight patients with anti-MDA5-positive DM died of RP-ILD, two died of RP-ILD combined with pulmonary infection, and the other two died of Kafka lung infections caused by pneumocystis. Subsequently, patients with anti-MDA5-positive DM were divided into two subgroups: non-survivors (*n* = 12) and survivors (*n* = 28). Compared to the survivors, more non-survivors had received short-term methylprednisolone impulse therapy (1,500 −2,500 mg, 33 vs. 4%, *p* = 0.022), antibiotics (83 vs. 36%, *p* = 0.014), and antivirals (75 vs. 32%, *p* = 0.018) for therapeutic or preventive objectives. There was no significant difference in using cyclophosphamide, JAK inhibitors, calcineurin inhibitors, or anti-fibrosis drugs between the two groups (all *p* > 0.05, Table 2).

**Table 2:** The medications for patients with anti-MDA5-positive DM during the 6-month follow-up.

**Medications**	**Total patients** **(***n*** = 40)**	**Non-survivors** **(***n*** = 12)**	**Survivors** **(***n*** = 28)**	***p***
Prednisone equivalent 1 ~ 2 mg/kg, *n* (%)	36 (90%)	12 (100%)	24 (86%)	0.421
Methylprednisolone impulse, 1,500 mg ~ 2500 mg, *n* (%)	5 (13%)	4 (33%)	1 (4%)	0.022
Cyclophosphamide, *n* (%)	29 (73%)	11 (92%)	18 (64%)	0.124
Mycophenolate mofetil, *n* (%)	3 (8%)	0	3 (11%)	N/A
JAK inhibitors, *n* (%)	6 (15%)	2 (17%)	4 (14%)	1.000
Calcineurin inhibitors, *n* (%)	17 (43%)	7 (58%)	10 (36%)	0.296
SMZ/TMP, *n* (%)	10 (25%)	3 (25%)	7 (25%)	1.000
Antibiotics, *n* (%)	20 (50%)	10 (83%)	10 (36%)	0.014
Antivirals, *n* (%)	18 (45%)	9 (75%)	9 (32%)	0.018
IVIG therapy, *n* (%)	19 (48%)	8 (67%)	11 (39%)	0.170
Anti-fibrosis treatment, *n* (%)	8 (20%)	2 (17%)	6 (21%)	1.000

*MDA5, melanoma differentiation-associated protein-5; DM, dermatomyositis; JAK, Janus kinase; SMZ/TMP, sulfamethoxazole or trimethoprim; IVIG, intravenous immunoglobulin; N/A, not applicable*.

### Clinical Features of Non-Survivors With Anti-MDA5-Positive DM

Compared baseline characteristics with the survivors, non-survivors with anti-MDA5-positive DM had advanced age of onset (59 ± 6 vs. 46 ± 9 years), higher rates of fever (75 vs. 18%) and positive CEA (75 vs. 14%), higher levels of serum ferritin (median 2,858 vs. 619 ug/L), LDH (median 472 vs. 309 U/L), and AST (median 59 vs. 32 U/L), but lower levels of hemoglobin (Hb, median 112 vs. 125 g/L) and ALB (median 25 vs. 32 g/L, all *p* < 0.05, Table 3 and Supplementary Table 3) at enrollment. Likewise, non-survivors with anti-MDA5-positive DM showed a significantly higher level of serum interleukin 2R (sIL2R), interleukin (IL)-6, and IL-10 than the survivors (all *p* < 0.05, Supplementary Table 3).

**Table 3:** Comparisons of clinical characteristics between non-survivors and survivors with anti-MDA5-positive DM.

**Characteristics**	**Patients with anti-MDA5-positive DM**		
	**Non-survivors** **(***n*** = 12)**	**Survivors** **(***n*** = 28)**	***p***
**Demographic**			
Female, *n* (%)	8 (67%)	17 (61%)	1.000
Age of onset (years), $\bar{x}$ ± s	59 ± 6	46 ± 9	<0.001
Disease course (months), $\bar{x}$ ± s	3.5 ± 2.9	3.6 ± 7.7	0.951
Smoking, *n* (%)	0 (0%)	7 (25%)	0.146
**General assessments**			
Fever, *n* (%)	9 (75%)	5 (18%)	0.002
Arthritis, *n* (%)	9 (75%)	22 (79%)	1.000
**Skin signs**			
Mechanic's hands, *n* (%)	5 (42%)	16 (57%)	0.494
Gottron sign/papules, *n* (%)	10 (83%)	21 (75%)	0.697
Heliotrope rash, *n* (%)	2 (17%)	4 (14%)	1.000
V-like sign, *n* (%)	5 (42%)	11 (39%)	1.000
Shawl sign, *n* (%)	3 (25%)	9 (32%)	0.725
**Muscle assessments**			
Muscle weakness, *n* (%)	4 (33%)	15 (54%)	0.311
Muscle pain, *n* (%)	3 (25%)	11 (39%)	0.484
Difficulty swallowing, *n* (%)	3 (25%)	3 (11%)	0.341
CADM, *n* (%)	6 (50%)	12 (43%)	0.677
**Pulmonary assessments**			
Cough, *n* (%)	7 (58%)	8 (29%)	0.091
Dyspnea, *n* (%)	7 (58%)	8 (29%)	0.091
ILD, *n* (%)	12 (100%)	26 (93%)	1.000
NSIP, *n* (%)	2 (17%)	16 (57%)	0.018
OP, *n* (%)	1 (8%)	5 (18%)	0.772
NSIP+OP, *n* (%)	6 (50%)	4 (14%)	0.046
UIP, *n* (%)	0 (0%)	1 (4%)	1.000
AIP, *n* (%)	3 (25%)	0 (0%)	0.022
HRCT-score	100 [80–140]	74 [62–121]	0.122
RP-ILD, *n* (%)	10 (83%)	4 (14%)	<0.001
**Laboratory examination**			
ALB (g/L), median (IQR)	25 [22–28]	32 [30–34]	<0.001
CK (U/L), median (IQR)	76 [37–242]	56 [36–135]	0.439
LDH (U/L), median (IQR)	472 [292–874]	307 [257–353]	0.009
ALT (U/L), median (IQR)	50 [29–78]	28 [16–59]	0.182
AST (U/L), median (IQR)	59 [45–118]	32 [20–58]	0.017
CRP (mg/dL), median (IQR)	1.2 [0.4–3.0]	0.3 [0.3–1.2]	0.060
Ferritin (ug/L), median (IQR)	2,858 [1,413–6,078]	619 [185–982]	0.016
KL-6^▴^ (U/ml), median (IQR)	1,462 [1,029–2,649]	1,075 [854–1,646]	0.377
Positive CEA, *n* (%)	9 (75%)	4 (14%)	0.001

▴ *The Krebs von den Lungen-6 (KL-6) was tested in five non-survivors and 11 survivors*.

*MDA5, melanoma differentiation-associated protein-5; DM, dermatomyositis; CADM, clinically amyopathic dermatomyositis; ILD, interstitial lung disease; NSIP, nonspecific interstitial pneumonia; OP, organizing pneumonia; UIP, usual interstitial pneumonia; AIP, acute interstitial pneumonia; HRCT, high-resolution computed tomography; RP-ILD, rapidly progressive ILD; ALB, albumin; CK, creatine kinase; LDH, lactate dehydrogenase; ALT, alanine aminotransferase; AST, aspartate aminotransferase; CRP, C-reactive protein; CEA, carcinoembryonic antigen; IQR, interquartile range*.

For pulmonary assessments, 58 (7/12) non-survivors and 7% (2/28) survivors were in severe condition at baseline that unable to complete either routine or bedside spirometry (*p* = 0.001). There were 100% of non-survivors and 93% of the survivors complicated with ILD at baseline (*p* > 0.05). Although the constituent ratio of pure NSIP was lower in the non-survivors (17 vs. 57%, *p* = 0.018), the constituent ratio of NSIP+OP in the non-survivors was significantly higher than that in the survivors (50 vs. 14%, *p* = 0.046). There was not any significant difference in cough, dyspnea, baseline HRCT-score, or the level of KL-6 between the two groups (all *p* > 0.05, Table 3). There were 83% of non-survivors developed RP-ILD during 6-month follow-up, significantly higher than the rate of the surviving control groups (83 vs. 14%, *p* < 0.001, Table 3). RP-ILD significantly contributed to the 6-month mortality in patients with anti-MDA5-positive DM (HR: 13.0, 95%CI: 2.8–60.1, *p* < 0.001).

### Baseline Risk Factors for 6-Month All-Cause Mortality in Patients With Anti-MDA5-Positive DM

For the early stratification of mortality risk in patients with anti-MDA5-positive DM before making therapeutic strategies, the potential mortality predictors were explored among baseline characteristics which were significantly different between non-survivors and survivors (Table 3 and Supplementary Table 3). The ROC curve showed that the age of onset, fever, serum ferritin, positive CEA, ALB, LDH, AST, Hb, sIL2R, IL-6, and IL-10 at baseline could significantly distinguish survivors and non-survivors, with the AUC values from 0.731 to 0.883 (all *p* < 0.05, Table 4). The corresponding cutoff values that were determined according to the optimal Youden indexes and the specificity no <75% are shown in Table 4.

**Table 4:** ROC curve analyses between non-survivors and survivors with anti-MDA5-positive DM.

**Characteristics**	**AUC**	**95%CI**	***p***	**Trade-off value**	**Sensitivity (%)**	**Specificity (%)**	
Age of onset (years)	0.876	0.770	0.983	<0.001	56 years	67%	89%
Fever	0.786	0.620	0.952	0.005	N/A
Ferritin (ug/L)	0.875	0.744	1.000	<0.001	1,250 ug/L	83%	89%
Positive CEA	0.804	0.640	0.967	0.003	N/A
ALB (g/L)	0.850	0.711	0.988	0.001	30 g/L	79%	92%
LDH (U/L)	0.759	0.589	0.929	0.010	368.5 U/L	67%	79%
AST (U/L)	0.738	0.577	0.899	0.018	56.5 U/L	67%	75%
Hb (g/L)	0.731	0.559	0.902	0.022	125 g/L	50%	92%
sIL2R (U/m)	0.864	0.675	1.000	0.011	1,572.5 U/ml	71%	100%
IL-6 (U/ml)	0.857	0.667	1.000	0.013	7.0 U/ml	71%	91%
IL-10 (U/ml)	0.883	0.725	1.000	0.008	6.7 U/ml	86%	82%

*ROC, receiver operating characteristic; AUC, area under curve; CI, confidence interval; CEA, carcinoembryonic antigen; ALB, albumin; LDH, lactate dehydrogenase; AST, aspartate aminotransferase; Hb, Hemoglobin; IL, interleukin; N/A, not applicable*.

**Figure 2:**
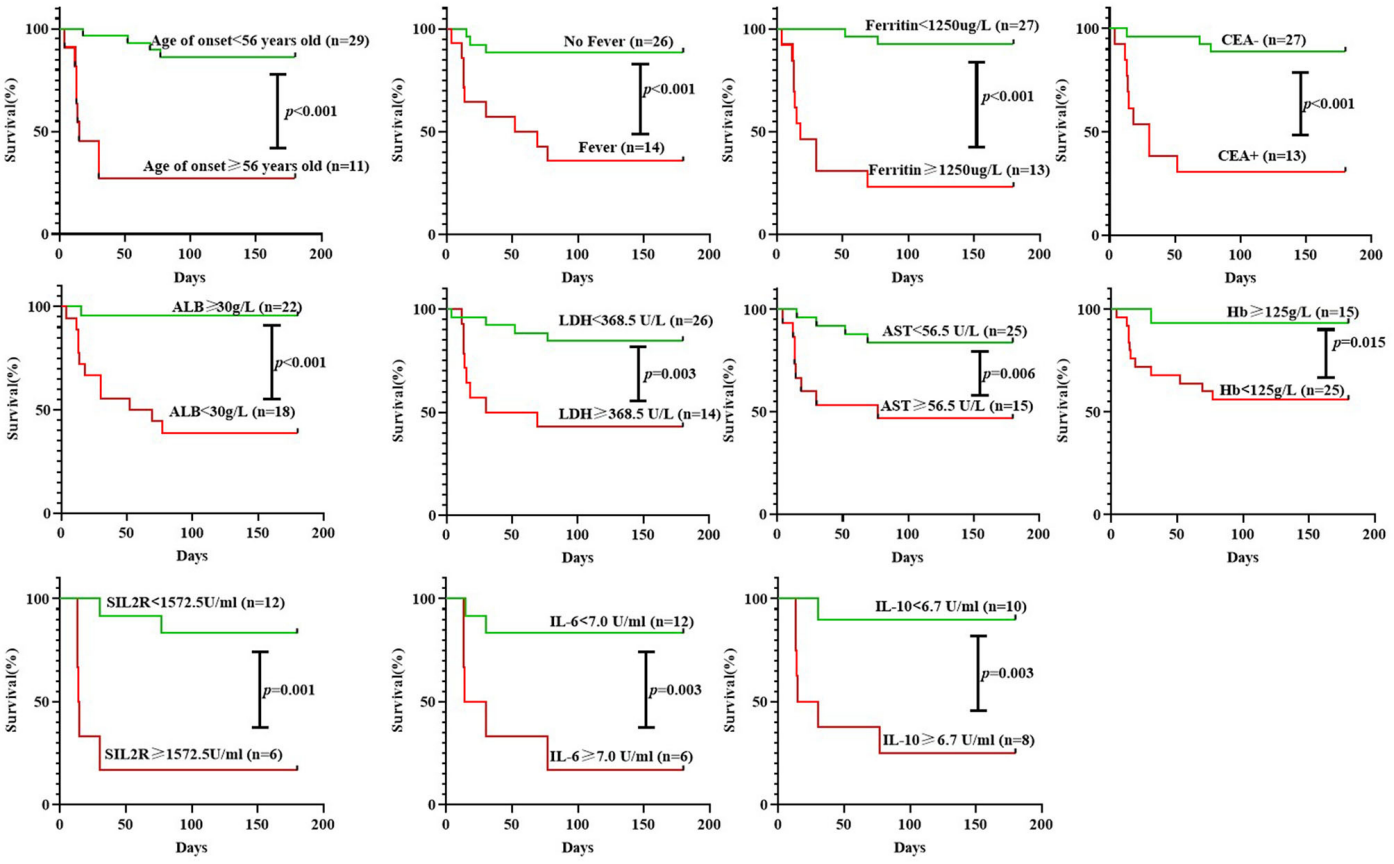
Survival curve analysis of 6-month all-cause mortality in patients with anti-MDA5-positive DM according to different groupings. CEA, carcinoembryonic antigen; LDH, lactate dehydrogenase; AST, aspartate aminotransferase; Hb, hemoglobin; ALB, albumin; IL, interleukin.

**Figure 3:**
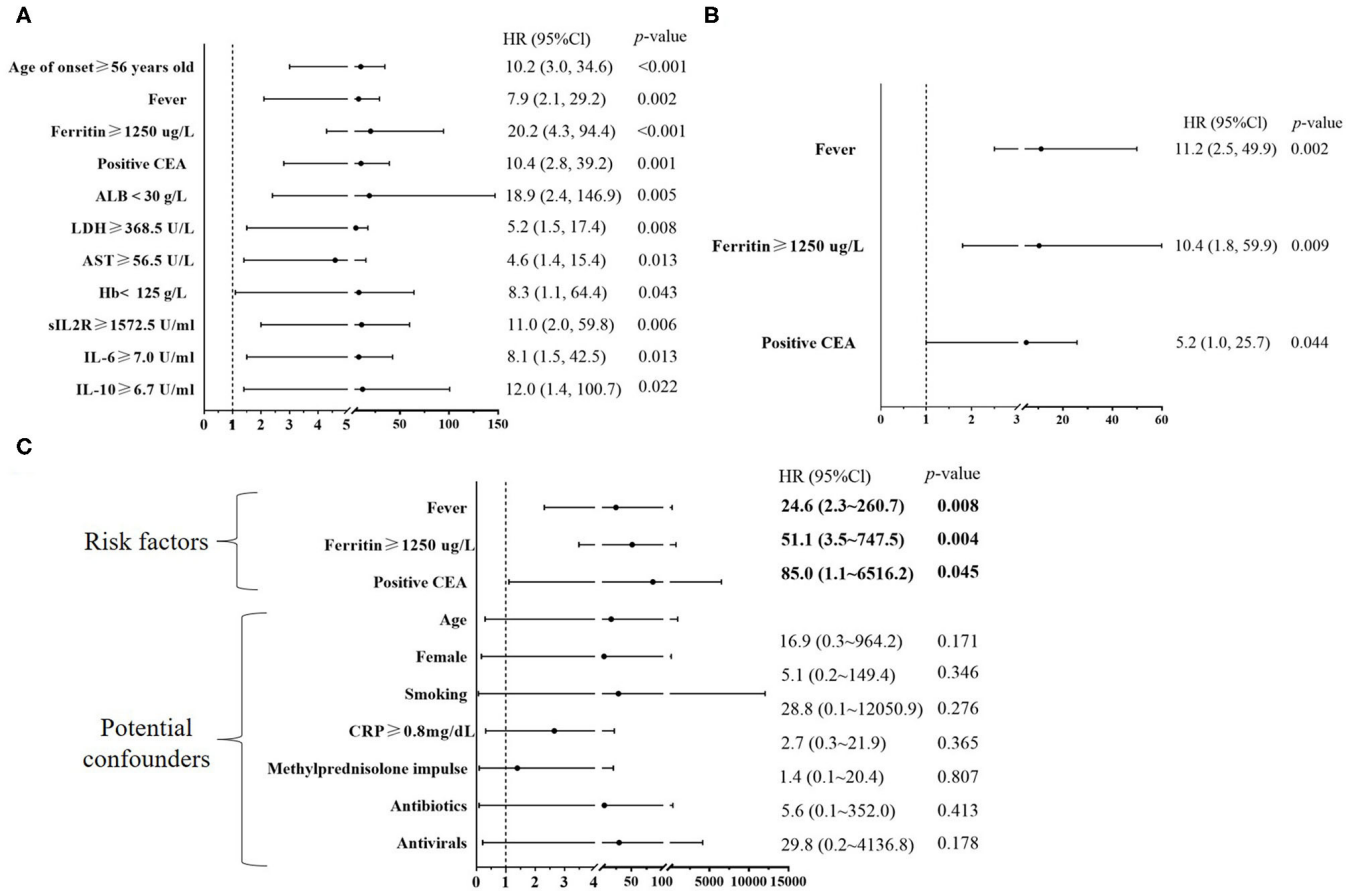
Baseline risk factors for six-month all-cause mortality in patients with anti-MDA5-positive DM. **(A)** A univariate Cox regression was performed to analyze baseline risk factors identified by the survival curve analysis of Figure 2; **(B)** A stepwise multivariate Cox regression was performed to analyze eight baseline risk factors including onset ≥ 56 years old, fever, serum ferritin ≥ 1,250 μg/L, positive carcinoembryonic antigen (CEA), albumin (ALB) < 30 g/L, lactate dehydrogenase (LDH) ≥ 368.5 U/L, aspartate aminotransferase (AST) ≥ 56.5 U/L, and hemoglobin (Hb)<125 g/L. The stepwise multivariate Cox regression followed the rule that variables were included when the *p*-value was < 0.05 or removed when the *p*-value was was >0.10. **(C)** Another multivariate Cox regression was used to adjust the potential confounders including age, sex, smoking, differential medications between the survivors and the non-survivors, and CRP ≥ 0.8 mg/dL. IL, interleukin.

Survival curve analysis showed that patients with the age of onset ≥ 56 years old, fever, serum ferritin ≥ 1,250 μg/L, positive CEA, ALB <30 g/L, LDH ≥ 368.5 U/L, AST ≥ 56.5 U/L, Hb <125 g/L, sIL2R ≥ 1,572.5U/ml, IL-6 ≥ 7.0U/ml, or IL-10 ≥ 6.7U/ml had significantly higher mortality than the corresponding controls, respectively (all *p* < 0.05, Figure 2). A univariate Cox regression showed the above 11 baseline characteristics were either risk factors for all-cause death within 6 months (all *p* < 0.05, Figure 3A). Except for sIL-2R, IL-6, or IL-10 which were tested in only 18 patients, the other eight risk factors were screened using the stepwise multivariate Cox regression following the rule that variables were included when the *p-*value was < 0.05 or removed when the *p-*value was >0.10. Finally, three of them could enter the equation as independent risk factors: fever (HR: 11.2, 95%CI: 2.5–49.9, *p* = 0.002), serum ferritin ≥ 1,250 μg/L (HR: 10.4, 95%CI: 1.8–59.9, *p* = 0.009), and positive CEA (HR: 5.2, 95%CI: 1.0–25.7, *p* = 0.044, Figure 3B). Next, another multivariate Cox regression was performed to adjust potential confounders including age, sex, smoking, differential medications between the survivors and the non-survivors, and CRP ≥ 0.8mg/dL which was recently reported as an independent risk factor (16), which showed that fever, ferritin ≥ 1,250 μg/L, and positive CEA were still independent risk factors for 6-month all-cause mortality in patients with anti-MDA5-positive DM (*p* < 0.05, Figure 3C).

### Matrix Prediction Model for 6-Month All-Cause Mortality in Patients With Anti-MDA5-Positive DM

**Figure 4:**
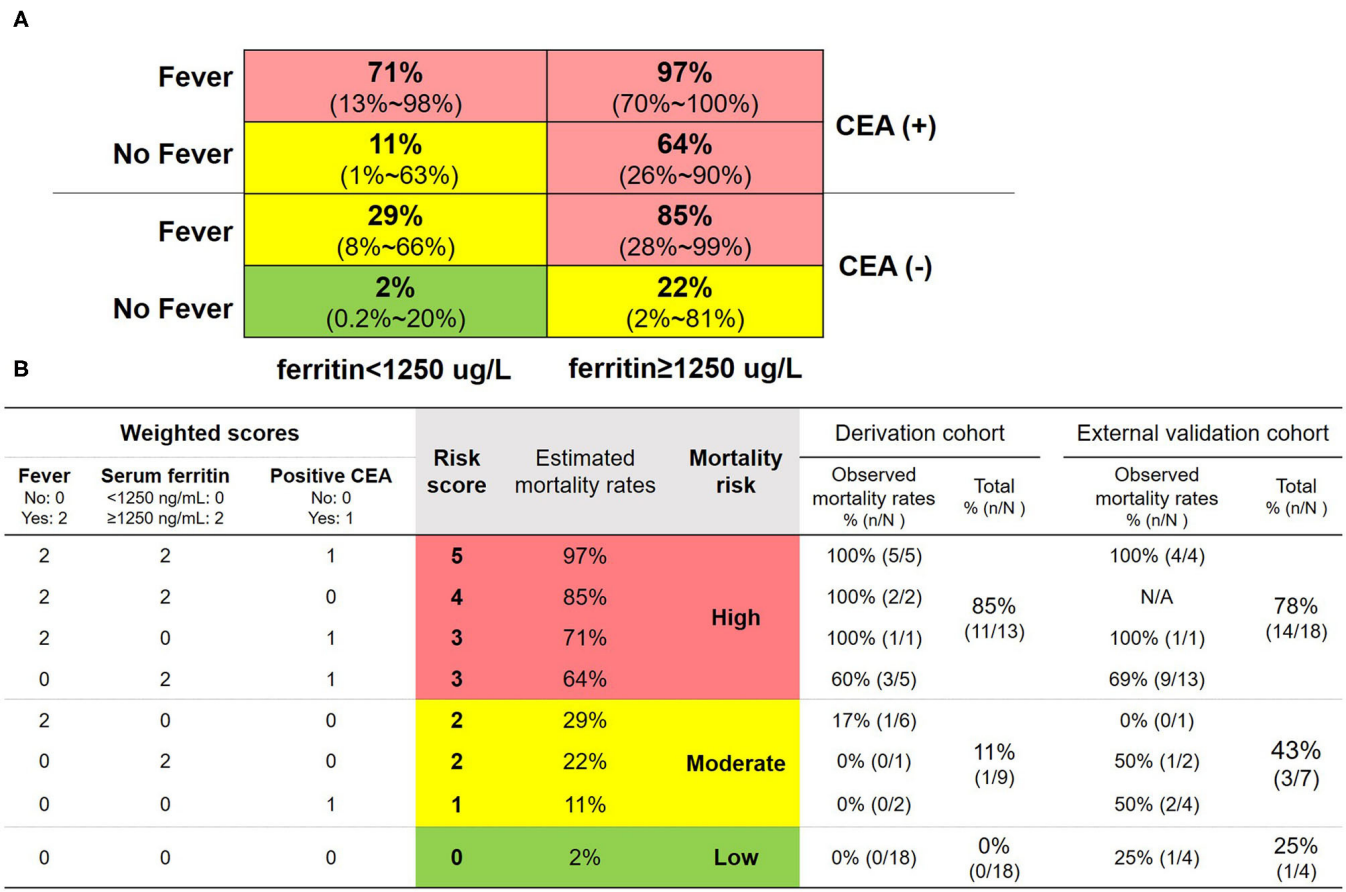
Matrix prediction model and the corresponding scoring system for 6-month all-cause mortality in patients with anti-MDA5-positive DM. **(A)** Matrix prediction model: fever yes or no, carcinoembryonic antigen (CEA) positive or negative, and ferritin <1,250 ug/L or ≥ 1,250 ug/L were combined to generate a matrix, and then, the relative mortality risk probability of death within 6 months in each grid was calculated. The 95% confidence intervals of the probabilities were shown in the parentheses. **(B)** Scoring system: three risk factors were weighted by regression coefficients, which were rounded into integer values. That is, fever scored 2 (yes) or 0 (no), serum ferritin scored 2 (≥1,250 μg/L) or 0 (<1,250 μg/L), and positive CEA scored 1 (yes) or 0 (no). The risk score was defined by adding the weighted scores of all three risk factors. Estimated and observed mortality rates of various combinations and two cohorts were shown. Red grids indicated high risk with the estimated mortality >50% or 3–5 risk scores. Yellow grids indicated moderate risk with the estimated mortality between 49–10% and 1–2 risk scores. Green grids indicated low risk with the estimated mortality <10% or 0 risk score.

Fever yes or no, CEA positive or negative, and serum ferritin < 1,250 ug/L or ≥ 1,250 ug/L were combined to generate a matrix, and then, the relative mortality risk probability within 6 months in each grid was calculated (Figure 4A). Accordingly, patients with anti-MDA5-positive DM could be stratified into three subgroups based on the various probabilities of estimated mortality: (i) high risk: A number of 13 (32.5%) patients with two or three of the above features (including fever, serum ferritin ≥ 1,250 μg/L, and positive CEA) had high estimated mortality with 97, 85, 71, and 64%, respectively, in four grids (in red, Figure 4A), and the total observed mortality was 85% (11/13, Figure 4B); (ii) moderate risk: A number of 9 (22.5%) patients with one of the above three features had moderate estimated mortality with 29, 22, and 11% in three grids (in yellow, Figure 4A), and the total observed mortality was 11% (1/9, Figure 4B); (iii) low risk: A number of 18 (45%) patients with none of the above three features had low estimated mortality with 2% (the green grid in Figure 4A), and the total observed mortality was 0% (0/18, = Figure 4B). To make the prognosis prediction of the model be more readily understood, the risk score was calculated based on the three risk factors weighted by regression coefficients, which were rounded into integer values. That is, fever scored 2 (yes) or 0 (no), serum ferritin scored 2 (≥ 1,250 μg/L) or 0 (<1,250 μg/L), and positive CEA scored 1 (yes) or 0 (no). The risk score was defined by adding the weighted values of all three risk factors. The estimated mortality rates of patients with the risk score of 0, 1, 2, 3, 4, and 5 were 2, 11, 22–29, 64–71, 85, and 97%, respectively. Therefore, 3–5 risk scores suggested high risk with the estimated mortality >50%; 1–2 risk scores suggested moderate risk with the estimated mortality between 49 and 10%; and 0 risk score indicated low risk with the estimated mortality <10% (Figure 4B).

### External Validation of the Matrix Prediction Model

A number of 40 patients with anti-MDA5-positive DM in another institution were used for the external validation (9, 10). The 6-month all-cause mortality was 63% (25/40) and all patients were complicated with ILD. A number of 11 patients were excluded because the data of serum ferritin at baseline were missing. Finally, 29 patients with anti-MDA5-positive DM were included for statistical analysis, with age 51 ± 9 years of onset and the disease course of DM at admission 4 ± 3 months, neither of which was significantly different from the derivation cohort (both *p* > 0.05, Table 5). Since the external validation cohort came from an institution famous for treating critical respiratory diseases, the 6-month all-cause mortality in the external validation was significantly higher than that in the derivation cohort (62 vs. 30%, *p* = 0.008). Likewise, there were 76% (22/29) of patients in the external validation cohort with positive CEA, significantly higher than that in the derivation cohort (*p* < 0.001, Table 5). There were 66% (19/29) of patients in the external validation cohort with serum ferritin ≥ 1,250 ug/L, and 21% (6/29) with fever, not significantly different than those in the derivation cohort. During 6-month follow-up, significantly more patients of the external validation cohort received aggressive treatment such as methylprednisolone impulse and IVIG therapy than the derivation cohort (both *p* < 0.05, Table 5). The top two commonly used immunosuppressants in the external validation cohort were also cyclophosphamide and calcineurin inhibitors, with a significantly higher usage rate of calcineurin inhibitors.

**Table 5:** Comparison of baseline characteristics and medications between two cohorts of patients with anti-MDA5-positive DM.

	**Derivation cohort** **(***n*** = 40)**	**External validation cohort** **(***n*** = 29)**	***p***
**Baseline Characteristics**			
Female, *n* (%)	25 (63%)	16 (55%)	0.541
Age of onset (years), $\bar{x}$ ± s	50 ± 10	51 ± 9	0.700
Disease course (months), $\bar{x}$ ± s	4 ± 7	4 ± 3	0.953
Smoking, *n* (%)	7 (18%)	6 (21%)	0.738
Fever, *n* (%)	14 (35%)	6 (21%)	0.196
**Laboratory examinations**			
ALB (g/L), median (IQR)	31 [26–33]	30 [25–32]	0.455
CK (U/L), median (IQR)	67 [36–135]	58 [23–170]	0.580
LDH (U/L), median (IQR)	314 [269–455]	366 [253–472]	0.851
CRP (mg/dL), median (IQR)	0.5 [0.3–1.8]	1.8 [0.4–4.4]	0.050
Ferritin (ug/L), median (IQR)	719 [379–2,305]	1370 [756–2,000]	0.228
KL-6 (U/ml), median (IQR)	1315 [8,89–1,754]^▴^	1,737 [702–2,611]	0.602
Positive CEA, *n* (%)	13 (33%)	22 (76%)	<0.001
**Medications during the 6-month follow-up**
Prednisone equivalent 1 ~ 2mg/kg, *n* (%)	36 (90%)	27 (93%)	0.985
Methylprednisolone impulse, *n* (%)	5 (13%)	22 (76%)	<0.001
Cyclophosphamide, *n* (%)	29 (73%)	20 (69%)	0.749
Mycophenolate mofetil, *n* (%)	3 (8%)	12 (41%)	0.001
JAK inhibitors, *n* (%)	6 (15%)	0	N/A
Calcineurin inhibitors, *n* (%)	17 (43%)	25 (86%)	<0.001
IVIG therapy, *n* (%)	19 (48%)	4 (14%)	0.003

▴ *The Krebs von den Lungen-6 (KL-6) was tested in 16 patients of the derivation cohort*.

*MDA5, melanoma differentiation-associated protein-5; DM, dermatomyositis; ALB, albumin; CK, creatine kinase; LDH, lactate dehydrogenase; CRP, C-reactive protein; CEA, carcinoembryonic antigen; IVIG, intravenous immunoglobulin; IQR, interquartile range*.

According to the matrix prediction model, 18 (62%) patients were high risk and the total observed mortality was 78% (14/18); seven (24%) patients were moderate risk and the total observed mortality was 43% (3/7); and four (14%) patients were low risk and the total observed mortality was 25% (1/4) in Figure 4B.

## Discussion

The current real-world observational cohort study built a novel matrix prediction model based on the three routine clinical characteristics (fever, serum ferritin, and CEA) in clinical practice rather than those based on the intricate algorithm from the published reports. Meanwhile, the application of baseline characteristics as mortality predictors can forecast the 6-month mortality as early as the moment of diagnosis of anti-MDA5-positive DM, which provides a chance for guiding the therapeutic options in clinical practice and optimizing subgroups enrichment in future trial designs. Additionally, we used a published cohort of patients with anti-MDA5-positive DM from another institution which validated the accuracy of this model (9, 10). The matrix model can provide the mortality risk probability in any combination of three features, which makes the prediction more accessible for clinicians and simpler to spread in clinical practice.

The prevalence of positive anti-MDA5 in patients with DM is 15 to 20% in Asia, which is higher than 4 to 7% in Europe and North America (23–25). Our study enrolled hospitalized patients and up to 49% of patients with DM had positive anti-MDA5, perhaps because patients with anti-MDA5-positive DM usually need hospitalization for intensive treatment. Consistent with the published data, Gottron sign/papules, arthritis, and ILD were independent associators with positive anti-MDA5 in our study. Our study showed the most common ILD pattern complicated with DM was NSIP including pure NSIP and NSIP+OP. It was reported that NSIP+OP pattern was strongly correlated with RP-ILD in patients with DM (26, 27). We showed that the ratio of NSIP+OP pattern was higher in anti-MDA5-positive DM than the negative control and was also higher in the non-survivors than the survivors among patients with anti-MDA5-positive DM, which suggests that the NSIP+OP pattern may correlate the poor outcome. Even though pure NSIP and pure OP usually respond well to glucocorticoid therapy, the response of NSIP+OP pattern to glucocorticoid therapy remains unclear and needs further study.

The outstanding feature of patients with anti-MDA5-positive DM is the extremely high mortality especially within the first half-year after disease onset (28, 29). The 6-month all-cause mortality in our study was 30%, consistent with other published real-world cohorts recruited by rheumatologists in various regions of China: 24% in Henan Province (11) and 41% in Shanghai City (9, 10). Considering the poor survival rate of the disease, the subgroup with high mortality risk is supposed to receive intensive treatment and care as early as possible, but it should be noted that methylprednisolone impulse therapy is not suitable. A recent study showed that non-survivors with anti-MDA5-positive DM received a significantly higher proportion of methylprednisolone impulse therapy than the survivor group (10). Likewise, in our study, among five patients with anti-MDA5-positive DM who received methylprednisolone impulse therapy, four patients in the high-risk group died of this aggressive therapy, while the other patients were in the moderate-risk group and survived. It suggests that methylprednisolone impulse therapy cannot save the fatalities in the high-risk subgroup. A reported FVC%-based model is expected to distinguish early-stage and advanced-stage patients of anti-MDA5-positive DM-ILD (9). A Japan cohort of 29 patients with anti-MDA5-positive DM at the early stage used combination therapy of high-dose glucocorticoids, tacrolimus, and intravenous cyclophosphamide, and the 6-month survival rate reached 89% (6). A recent single-centered open-labeled trial found that a routine dose of tofacitinib combined with glucocorticoid could significantly improve the survival of early-stage patients with amyopathic DM-associated ILD (30). The novel matrix prediction model in this study is also helpful for the risk stratification in patients with anti-MDA5-positive DM. Further subgroup cohort studies are needed for exploring the effectiveness and safety profile of these treatment protocols for patients with various probabilities of mortality risk predicted by our matrix model.

The elevation of ferritin in the serum of patients with anti-MDA5-positive DM may indicate the hyperinflammatory state of severe disease. Serum ferritin was first described in 2010 as the most significant prognostic factor for the mortality prediction in a Japanese cohort of patients with anti-MDA5-positive DM complicated with ILD (31). This study also suggested that elevation of ferritin (≥500 ng/ml) portended decreased survival, and the strength of this association increased with increasing ferritin levels (≥1,600 ng/ml) (31). However, a recent study failed to show serum ferritin associated with the prognosis of anti-MDA5-positive DM because most of the patients missed the ferritin tests (14). A number of 11 patients in our external validation cohort were excluded also because of missed ferritin tests. To highlight the importance of this outstanding risk factor in patients with anti-MDA5-positive DM, our study used the optimal Youden indexes to determine a cutoff value of 1,250 ng/ml, refresh the role of serum ferritin for independent mortality prediction by multivariate regression, and further included it in a novel matrix prediction model. It would help to remind clinicians to test serum ferritin when they evaluate the disease severity of patients with anti-MDA5-positive DM.

Our study showed 32% of patients with DM and 35% of patients with anti-MDA5-positive DM presented with fever at baseline before the treatment of DM, while 75% of non-survivors with anti-MDA5-positive DM, significantly higher than the survivors (18%); and fever was one of three independent mortality risk factors in the matrix prediction model. It was reported that the prevalence of fever in DM patients was 2.4–23.8% (32, 33). Infection is reported to be one of the most important risk factors for mortality in patients with DM (34–36). However, non-survivors with anti-MDA5-positive DM in our study received a significantly higher proportion of antibiotic and antiviral drugs than the survivor group, which suggests that the therapy pointing to pathogenic microorganisms cannot change the outcome of patients with high mortality risk. Fever at the onset of DM may indicate not only the existence of concomitant infection (32, 37) but also the non-infectious hyperinflammatory state.

Even though anti-MDA5-positive DM is not associated with an increased risk of malignancy (0–4%) (38), an interesting finding in our study is that 33% of patients with anti-MDA5-positive DM in the derivation cohort and 76% of the validation cohort showed slight elevation of CEA, and positive CEA was the independent risk factor for 6-month mortality. Besides a well-known biomarker for adenocarcinoma, the serum CEA was also correlated with the severity of idiopathic pulmonary fibrosis (39–41) and serves as a powerful indicator of RP-ILD and poor prognosis in patients with CADM (42). It was speculated that CEA may bind with the receptor on macrophages or Kupffer cells to cause cell activation and cytokine production in patients with CADM (43), so the hyperinflammatory state of DM may also result in elevated CEA. During the extended follow-up for the patients with DM with positive CEA after our study, no adenocarcinoma was confirmed and the elevated CEA decreased to normal as the remission of DM (data not shown).

There are three major limitations in this study. First, our study suggested three clinical features as the independent risk factors of 6-month mortality in patients with anti-MDA5-positive DM and all of them may reflect the hyperinflammatory state of DM. Consistently, inflammatory cytokines such as sIL2R, IL-6, and IL-10 were significantly correlated with mortality risk according to the univariate Cox regression analyses. However, they were tested in less than half of patients with anti-MDA5-positive DM and therefore were excluded from the development of the prediction model due to the small sample size. Inflammatory cytokine assessments would be highlighted in further studies of patients with DM especially those with anti-MDA5-positive, to provide more evidence of its effects. Second, anti-MDA5-positive DM is a rare autoimmune disease, so we spent 37 months on prospectively recruiting 40 patients with anti-MDA5-positive DM. The sample size of other single-center studies was usually around 40 patients or less (12, 14, 29, 44–47), except for a retrospective multicenter Japanese cohort (16, 48), a multicenter Chinese cohort (9), and a retrospective single-center Chinese cohort (11). To validate the result, we retrospectively analyzed a published cohort of 40 patients from another institution as the external validation, but a pity that 11 patients were excluded because the data of serum ferritin at baseline were missing. The sample size of moderate- or low-risk group in the validation cohort was small. It would be necessary to carry out further multicenter studies with a higher proportion of moderate- and low-risk groups to verify the strength of this model. Third, our study is designed as a real-world observational study, and further molecular mechanism research of our findings including fever, ferritin, CEA, and inflammatory cytokines would be needed in the future to investigate their links to DM disease development.

In conclusion, baseline characteristics such as fever, ferritin ≥ 1,250 μg/L, and positive CEA are the independent risk factors for 6-month all-cause mortality in patients with anti-MDA5-positive DM. This study emphasizes the importance of ferritin and CEA testing in patients with anti-MDA5-positive DM, although they have not been paid attention to in clinical practice previously. A novel matrix prediction model composed of these three routine baseline clinical indicators is first proposed for clinicians making initiate therapeutic decision. This model provides a chance for exploration of individual treatment strategies in anti-MDA5-positive DM subgroups with various probabilities of mortality risk.

## Data Availability Statement

The original contributions presented in the study are included in the article/Supplementary Material, further inquiries can be directed to the corresponding authors.

## Ethics Statement

The Medical Ethics Committee of Sun Yat-sen Memorial Hospital approved the protocol (SYSEC-KY-KS-2021-248). The patients/participants provided their written informed consent to participate in this study.

## Author Contributions

Z-MO and J-ZL contributed equally to this work, including conceiving and designing the study, reading and analyzing documents, performing the statistical analysis, and drafting the manuscript. Corresponding authors QH, LD, and Y-QM also conceived and participated in its design, advised on the search, read and analyzed documents, and edited the article. A-JT and L-JY participated in qualitatively and quantitatively anti-MDA5 determination in patients with DM and critically revised the manuscript. Z-HY carried out the radiographic assessment and critically revised the manuscript. X-NW, Q-HL, J-JL, and D-HZ participated in the clinical assessment of patients with DM in derivation cohort and critically revised the manuscript. B-PG and GZ participated in clinical assessment of DM patients in validation cohort. All authors read and approved the final manuscript.

## Funding

This work was supported by the National Natural Science Foundation of China (Grant No. 82101892), Guangdong Basic and Applied Basic Research Foundation (Grant No. 2020A1515110061), Project funded by China Postdoctoral Science Foundation (Grant No. 2021M703722), and Guangdong Medical Scientific Research Foundation (Grant No. A2021065).

## Conflict of Interest

The authors declare that the research was conducted in the absence of any commercial or financial relationships that could be construed as a potential conflict of interest.

## Publisher's Note

All claims expressed in this article are solely those of the authors and do not necessarily represent those of their affiliated organizations, or those of the publisher, the editors and the reviewers. Any product that may be evaluated in this article, or claim that may be made by its manufacturer, is not guaranteed or endorsed by the publisher.
