# Supplementary material for: A Matrix Prediction Model for the 6-Month Mortality Risk in Patients With Anti-Melanoma Differentiation-Associated Protein-5-Positive Dermatomyositis
Source: Front Med (Lausanne). 2022 Apr 1;9:860798. doi: 10.3389/fmed.2022.860798 (PMC9010999; doi:10.3389/fmed.2022.860798)
Supplement: Supplementary file 1 [file Table_1.docx]

**Supplementary table 1 Clinical characteristics of 82 DM patients and the subgroups based on the anti-MDA5 (Continuation of Table 1)**

| **Characteristics** | **DM patients (n=82)** | **DM patients** | | |
| --- | --- | --- | --- | --- |
|  |  | **Anti-MDA5-Positive (n=40)** | **Anti-MDA5-Negative (n=42)** | ***p*** |
| **Blood routine examination** |  |  |  |  |
| Platelets (×10^12^/L), median (IQR) | 252 [187-327] | 229 [151-285] | 282 [232-343] | **0.007** |
| Hemoglobin (g/L), median (IQR) | 123 [111-132] | 120 [108-130] | 126 [115-138] | 0.060 |
| WBC (×10^9^/L), median (IQR) | 7 [5-9] | 6 [4-8] | 7 [6-11] | **0.001** |
| Lymphocytes (×10^9^/L), median (IQR) | 1.0 [0.6-1.4] | 0.9 [0.5-1.3] | 1.1 [0.7-1.5] | 0.160 |
| Neutrophils (×10^9^/L), median (IQR) | 4.6 [3.5-7.2] | 4.0 [2.4-5.9] | 5.8 [4.1-8.9] | **0.001** |
| **Immunological indicators** |  |  |  |  |
| GLB (g/L), median (IQR) | 34 [29-39] | 33 [29-38] | 34 [29-40] | 0.802 |
| C3 (mg/L), median (IQR) | 1070 [922-1185] | 1070 [942-1170] | 1080 [899-1195] | 0.788 |
| C4 (mg/L), median (IQR) | 247 [200-321] | 289 [219-367] | 232 [185-297] | **0.005** |
| ANA, n (%) | 57 (70%) | 26 (65%) | 31 (74%) | 0.386 |
| Anti-SSA, n (%) | 19 (23%) | 7 (18%) | 12 (29%) | 0.235 |
| Anti-Ro52, (%) | 51 (62%) | 28 (70%) | 23 (55%) | 0.155 |
| **Indicators of infection and inflammation** |  |  |  |  |
| Positive T-SPOT, n (%) | 9 (11%) | 5 (13%) | 4 (10%) | 0.666 |
| EBV infection^#^, n (%) | 47 (57%) | 18 (45%) | 29 (69%) | **0.028** |
| CMV infection^&^, n (%) | 9 (11%) | 3 (8%) | 6 (14%) | 0.529 |
| ESR (mm/h), median (IQR) | 38 [16-60] | 42 [19-61] | 29 [11-55] | 0.139 |
| **Lymphocyte subsets**^▲^ |  |  |  |  |
| CD3+T cells (%), median (IQR) | 67 [60-77] | 64 [54-75] | 69 [62-78] | 0.343 |
| CD3-CD19+B cells (%), median (IQR) | 16 [13-25] | 19 [14-30] | 15 [7-24] | 0.101 |
| NK cells (%), median (IQR) | 9 [5-20] | 8 [5-13] | 11 [5-24] | 0.550 |
| CD4+T cells (%), median (IQR) | 39 [33-48] | 41 [32-52] | 37 [33-48] | 0.737 |
| CD8+T cells (%), median (IQR) | 23 [15-32] | 22 [12-29] | 24 [17-35] | 0.651 |
| CD4+T /CD8+T cells, median (IQR) | 2.0 [0.9-2.8] | 2.3 [1.2-3.1] | 1.4 [0.9-2.7] | 0.431 |
| CD3+CD56+ cells (%), median (IQR) | 0.7 [0.3-0.9] | 0.5 [0.2-0.9] | 0.7 [0.4-1.0] | 0.533 |
| **Serum cytokines^△^** |  |  |  |  |
| sIL2R (U/ml), median (IQR) | 1073 [648-1426] | 1222 [726-1621] | 823 [395-1207] | 0.111 |
| IL-6 (pg/ml), median (IQR) | 4.6 [2.5-12.3] | 4.6 [3.6-9.9] | 4.3 [2.1-14.8] | 0.425 |
| IL-8 (pg/ml), median (IQR) | 24.0 [17.2-58.4] | 20.2 [14.0-60.4] | 35.5 [22.9-62.3] | 0.149 |
| IL-10 (pg/ml), median (IQR) | 5.5 [5.0-10.0] | 5.8 [5.0-13.6] | 5.0 [5.0-6.6] | 0.148 |
| IL-1 (pg/ml), median (IQR) | 5.0 [5.0-5.5] | 5.0 [5.0-5.6] | 5.0 [5.0-5.5] | 1.000 |
| TNF-α (pg/ml), median (IQR) | 13.0 [10.7-19.7] | 14.1 [11.2-22.4] | 12.3 [8.9-19.2] | 0.482 |

# EBV infection was defined as EBV DNA in peripheral blood≥1.0×10^3^.

& CMV infection was defined as CMV DNA in peripheral blood≥1.0×10^3^.

▲ Lymphocyte subsets were tested in 19 anti-MDA5-positive DM patients and 19 anti-MDA5-negative DM patients, a total of 38 DM patients.

**△** Serum cytokines were tested in 18 anti-MDA5-positive DM patients and 14 anti-MDA5-negative DM patients, a total of 32 DM patients.

MDA5, melanoma differentiation-associated protein-5; DM, dermatomyositis; WBC, white blood cells; GLB, globulin; ANA, anti-nuclear antibody; anti-SSA, anti-Sjogren's-syndrome-related antigen A; T-SPOT, T cell spot test; EBV, epstein-barr virus; CMV, cytomegalovirus; ESR, erythrocyte sedimentation rate; NK, natural killer; IL, interleukin; TNF, tumor necrosis factor; IQR, interquartile range.
