# Supplementary material for: A Matrix Prediction Model for the 6-Month Mortality Risk in Patients With Anti-Melanoma Differentiation-Associated Protein-5-Positive Dermatomyositis
Source: Front Med (Lausanne). 2022 Apr 1;9:860798. doi: 10.3389/fmed.2022.860798 (PMC9010999; doi:10.3389/fmed.2022.860798)
Supplement: Supplementary file 2 [file Table_2.docx]

**Supplementary table 2 Univariate and multivariate logistic regression analyses showed the associators with positive anti-MDA5 in 82 DM patients**

| **Characteristics** | **Univariate analysis** | | |  | **Multivariate analysis^#^** | | |
| --- | --- | --- | --- | --- | --- | --- | --- |
|  | ***p*** | **OR** | **95% CI** |  | ***p*** | **OR** | **95% CI** |
| Disease course (months) | 0.003 | 1.009 | 1.003~1.015 |  |  |  |  |
| Arthritis | <0.001 | 7.684 | 2.857~20.664 |  | 0.011 | 5.184 | 1.455~18.467 |
| Gottron sign/papules | <0.001 | 5.597 | 2.214~14.748 |  | 0.010 | 5.135 | 1.489~17.708 |
| ILD | 0.001 | 12.920 | 2.743~60.852 |  | 0.034 | 7.034 | 1.157~42.785 |
| CK (U/L) | 0.045 | 1.000 | 1.000~1.001 |  |  |  |  |
| Ferritin (ug/L) | 0.045 | 1.000 | 1.000~1.001 |  |  |  |  |
| C4 (mg/L) | 0.003 | 1.009 | 1.003~1.015 |  | 0.014 | 1.010 | 1.002~1.017 |
| Positive CEA | 0.008 | 6.259 | 1.626~24.093 |  |  |  |  |
| Platelets (×10^12^/L) | 0.025 | 0.995 | 0.990~0.999 |  |  |  |  |
| WBC (×10^9^/L) | 0.008 | 0.828 | 0.720~0.952 |  |  |  |  |
| Neutrophils (×10^9^/L) | 0.006 | 0.797 | 0.677~0.937 |  |  |  |  |
| EBV | 0.030 | 0.367 | 0.149~0.905 |  |  |  |  |

# The variables listed in the univariate analysis were entered into multivariate analysis.

MDA5, melanoma differentiation-associated protein-5; DM, dermatomyositis; ILD, interstitial lung disease; CEA, carcinoembryonic antigen; WBC, white blood cells; CK, creatine kinase; OR, odds ratio; CI: confidence interval.
