# Supplementary material for: A Matrix Prediction Model for the 6-Month Mortality Risk in Patients With Anti-Melanoma Differentiation-Associated Protein-5-Positive Dermatomyositis
Source: Front Med (Lausanne). 2022 Apr 1;9:860798. doi: 10.3389/fmed.2022.860798 (PMC9010999; doi:10.3389/fmed.2022.860798)
Supplement: Supplementary file 3 [file Table_3.docx]

**Supplementary table 3 Comparisons of clinical characteristics between non-survivors and survivors with anti-MDA5-positive DM (Continuation of Table 3)**

| **Characteristics** | **Anti-MDA5-positive DM patients** | | |
| --- | --- | --- | --- |
|  | **Non-survivors (n=12)** | **Survivors (n=28)** | ***p*** |
| **Blood routine examination** |  |  |  |
| Platelets (×10^12^/L), median (IQR) | 158 [105-306] | 239 [160-285] | 0.122 |
| Hemoglobin (g/L), median (IQR) | 112 [78-122] | 125 [113-131] | **0.021** |
| WBC (×10^9^/L), median (IQR) | 7 [4-9] | 6 [3-8] | 0.439 |
| Lymphocytes (×10^9^/L), median (IQR) | 0.7 [0.4-1.0] | 1.1 [0.5-1.4] | 0.074 |
| Neutrophils (×10^9^/L), median (IQR) | 4.3 [2.5-6.8] | 4.0 [2.4-5.0] | 0.512 |
| **Immunological indicators** |  |  |  |
| GLB (g/L), median (IQR) | 33 [30-38] | 33 [28-37] | 0.652 |
| C3 (mg/L), median (IQR) | 1105 [871-1237] | 1040 [952-1140] | 1.000 |
| C4 (mg/L), median (IQR) | 318 [228-465] | 265 [219-341] | 0.233 |
| ANA, n (%) | 9 (75%) | 17 (61%) | 0.484 |
| Anti-SSA, n (%) | 2 (17%) | 5 (18%) | 1.000 |
| Anti-Ro52, n (%) | 9 (75%) | 19 (68%) | 0.725 |
| **Indicators of infection and inflammation** |  |  |  |
| Positive T-SPOT, n (%) | 2 (17%) | 3 (11%) | 0.627 |
| EBV infection^#^, n (%) | 5 (42%) | 13 (46%) | 1.000 |
| CMV infection^&^, n (%) | 1 (8%) | 2 (7%) | 1.000 |
| ESR (mm/h), median (IQR) | 55 [38-78] | 37 [17-53] | 0.069 |
| **Lymphocyte subsets**^▲^ |  |  |  |
| CD3+T cells (%), median (IQR) | 61 [42-80] | 68 [58-74] | 0.902 |
| CD3-CD19+B cells (%), median (IQR) | 23 [14-33] | 19 [14-28] | 0.650 |
| NK cells (%), median (IQR) | 8 [5-20] | 10 [6-13] | 0.837 |
| CD4+T cells (%), median (IQR) | 39 [32-52] | 42 [31-52] | 0.592 |
| CD8+T cells (%), median (IQR) | 22 [10-27] | 22 [13-34] | 0.773 |
| CD4+T /CD8+T cells, median (IQR) | 2.4 [1.2-3.6] | 2.3 [0.9-3.0] | 0.873 |
| CD3+CD56+ cells (%), median (IQR) | 0.7 [0.4-1.0] | 0.5 [0.2-0.9] | 0.529 |
| **serum cytokines^△^** |  |  |  |
| sIL2R (U/ml), median (IQR) | 1727 [1210-2259] | 785 [629-1270] | **0.008** |
| IL-6 (pg/ml), median (IQR) | 12.3 [4.6-53.0] | 4.0 [2.5-5.2] | **0.011** |
| IL-8 (pg/ml), median (IQR) | 23.6 [19.2-64.7] | 16.0 [12.6-59.0] | 0.126 |
| IL-10 (pg/ml), median (IQR) | 13.0 [7.6-36.2] | 5.0 [5.0-5.9] | **0.006** |
| IL-1 (pg/ml), median (IQR) | 5.0 [5.0-5.5] | 5.0 [5.0-8.3] | 0.860 |
| TNF-α (pg/ml), median (IQR) | 19.7 [11.2-23.5] | 13.6 [11.0-16.6] | 0.285 |

# EBV infection was defined as EBV DNA in peripheral blood≥1.0×10^3^.

& CMV infection was defined as CMV DNA in peripheral blood≥1.0×10^3^.

▲ Lymphocyte subsets were tested in 7 non-survivors and 12 survivors.

△ serum cytokines were tested in 7 non-survivors and 11 survivors.

MDA5, melanoma differentiation-associated protein-5; DM, dermatomyositis; WBC, white blood cells; GLB, globulin; ANA, anti-nuclear antibody; anti-SSA, anti-Sjogren's-syndrome-related antigen A; T-SPOT, T cell spot test; EBV, epstein-barr virus; CMV, cytomegalovirus; ESR, erythrocyte sedimentation rate; NK, natural killer; IL, interleukin; TNF, tumor necrosis factor; IQR, interquartile range.
